# Cytotoxic Effects of Cannabidiol on Neonatal Rat Cortical Neurons and Astrocytes: Potential Danger to Brain Development

**DOI:** 10.3390/toxins14100720

**Published:** 2022-10-21

**Authors:** Damijana Mojca Jurič, Klara Bulc Rozman, Metoda Lipnik-Štangelj, Dušan Šuput, Miran Brvar

**Affiliations:** 1 Institute of Pharmacology and Experimental Toxicology, Faculty of Medicine, University of Ljubljana, Korytkova 2, 1000 Ljubljana, Slovenia; 2 Institute of Pathophysiology, Faculty of Medicine, University of Ljubljana, Zaloška Cesta 4, 1000 Ljubljana, Slovenia; 3 Centre for Clinical Physiology, Faculty of Medicine, University of Ljubljana, Zaloška Cesta 4, 1000 Ljubljana, Slovenia; 4 Centre for Clinical Toxicology and Pharmacology, University Medical Centre Ljubljana, Zaloška Cesta 7, 1000 Ljubljana, Slovenia

**Keywords:** cannabidiol, astrocytes, neurons, perinatal, cytotoxicity, apoptosis

## Abstract

The influence of cannabidiol (CBD) on brain development is inadequately understood. Since CBD is considered a non-intoxicating drug, it has attracted great interest concerning its potential medical applicability, including in pregnant women and children. Here, we elucidated the response of perinatal rat cortical neurons and astrocytes to CBD at submicromolar (0.1, 0.5, 1, 5 µM) concentrations attainable in humans. The effect of CBD was concentration- and time-dependent and cell-specific. In neurons, 0.1 µM CBD induced an early and transient change in mitochondrial membrane potential (ΔΨm), ATP depletion, and caspase-8 activation, followed by rapid ATP recovery and progressive activation of caspase-9 and caspase-3/7, resulting in early apoptotic cell death with reduction and shortening of dendrites, cell shrinkage, and chromatin condensation. The decrease in neuronal viability, ATP depletion, and caspase activation due to CBD exposure was prevented by transient receptor potential vanilloid 1 (TRPV1) antagonist. In astrocytes, 0.5 µM CBD caused an immediate short-term dysregulation of ΔΨm, followed by ATP depletion with transient activation of caspase-8 and progressive activation of caspase-9 and caspase-3/7, leading to early apoptosis and subsequent necroptosis. In astrocytes, both TRPV1 and cannabinoid receptor 1 (CB_1_) antagonists protected viability and prevented apoptosis. Given that CBD is a non-intoxicating drug, our results clearly show that this is not the case during critical periods of brain development when it can significantly interfere with the endogenous cannabinoid system.

## 1. Introduction

The cerebral cortex is a complex brain structure that coordinates sensory and motor information and enables the performance of many higher-level cognitive functions. Correct establishment of this structure is critically dependent on dynamic interactions of neurons and glia during development [1,2,3,4]. The endogenous cannabinoid system (ECS) is an important brain modulatory network with a broad regulatory role throughout all stages of development, such as neurogenesis, glial formation, neuronal migration, synaptogenesis, and regulation of signalling pathways and synaptic transmission [5]. The ECS comprises cannabinoid receptors 1 and 2 (CB_1_ and CB_2_), endogenous ligands, including the most studied 2-arachidonoylglycerol (2-AG) and anandamide (AEA), and endocannabinoid metabolic enzymes [6]. The ECS was discovered after cannabinoid receptors were identified as primary targets of compounds present in psychotropic preparations of the *Cannabis sativa* plant. The widespread legalization of cannabis for recreational and medical use has dramatically increased the potential health risk for vulnerable populations in recent years. Various studies indicate that exposure to Δ^9^-tetrahydrocannabinol (Δ^9^-THC), the main intoxicating component of cannabis and a relatively specific CB_1_ receptor agonist, during the prenatal/perinatal and adolescent period could alter the pathways of many neurobiological systems and consequently impair synaptic plasticity and behavioural phenotypes in the adult brain [7,8].

The influence of cannabidiol (CBD), the main non-psychoactive phytocannabinoid, on brain development is inadequately understood. Being considered a non-intoxicating drug, CBD has attracted a great deal of interest for its potential broad medical applicability [9,10,11]. Unlike Δ^9^-THC, the pharmacology of CBD is complex and highly variable. It can modulate the activity of several non-canonical receptors, transporters, and enzymes in addition to cannabinoid receptors [12]. Some effects of CBD on these targets in vitro are only manifested at high concentrations [13], which may be difficult to achieve in vivo, specifically due to CBD’s relatively poor bioavailability [14,15,16]. In the particular study, we sought to elucidate the response of perinatal rat cortical neurons and astrocytes to CBD at submicromolar concentrations attainable in humans. Impaired mitochondria function, ATP depletion, reduced viability, and a significant rate of cell death due to CBD exposure were observed in both neural cell types. Our findings suggest that not only Δ^9^-THC [7,8] but also CBD may impair cortical brain cells during critical periods of development in concentration, time, and in a cell-specific manner, with differential regulation of processes that could contribute to cell reprogramming and cause cell death.

## 2. Results and Discussion

**Figure 1:**
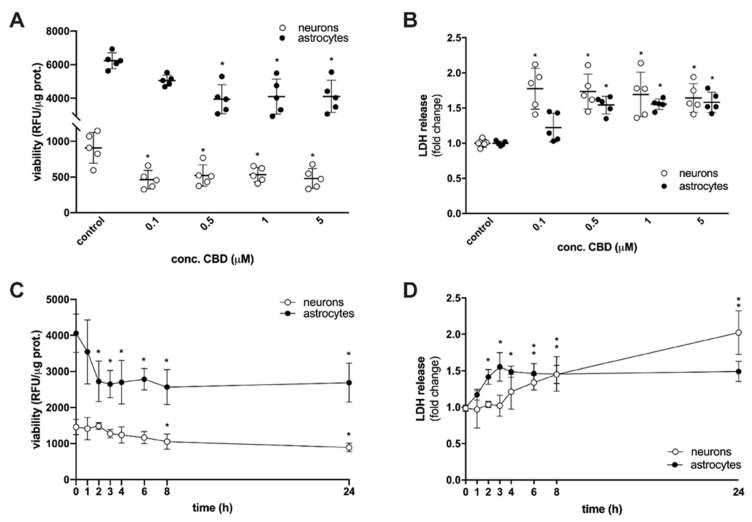
CBD decreases viability of rat cortical neurons and astrocytes in a concentration and time-dependent manner. (**A**) Cells were treated with 0.1, 0.5, 1, or 5 µM CBD for 24 h. Cell viability was evaluated by resazurin-based metabolic assay; data (relative fluorescence units-RFU) were normalized to protein concentration and shown as mean ± SD (*n* = 5). Two-way ANOVA [F_4,40_ = 5.278; *p* = 0.0016] followed by Tukey’s post hoc test, * *p* < 0.05 vs. control. (**B**) Cells were treated with 0.1, 0.5, 1, or 5 µM CBD for 24 h. Levels of non-viable cells were estimated by measuring LDH release; data were normalized to the mean value of the control and shown as mean ± SD (*n* = 5). Two-way ANOVA [F_4,40_ = 3.11; *p* = 0.0256] followed by Tukey’s post hoc test, * *p* < 0.05 vs. control. (**C**) Neurons were treated with 0.1 µM CBD and astrocytes were treated with 0.5 µM CBD for 1 to 24 h. Cell viability was evaluated by resazurin-based metabolic assay; data (relative fluorescence units-RFU) were normalized to protein concentration and shown as mean ± SD (*n* = 5). Two-way ANOVA [F_7,64_ = 2.886; *p* = 0.0110] followed by Tukey’s post hoc test, * *p* < 0.05 vs. control. (**D**) Neurons were treated with 0.1 µM CBD and astrocytes were treated with 0.5 µM CBD for 1 to 24 h. Levels of non-viable cells were estimated by measuring LDH release; data were normalized to the mean value of the control and shown as mean ± SD (*n* = 5). Two-way ANOVA [F_7,64_ = 9.8; *p* < 0.0001] followed by Tukey’s post hoc test, * *p* < 0.05 vs. control.

Recent comprehensive reviews on pharmacokinetic parameters in humans following CBD administration revealed that pharmacologically relevant dosing of CBD, depending on the four main routes of administration, ranges from nanomolar to, at most, the 2–3 µM range [6,15]. To ascertain whether concentrations attainable in humans hamper brain cells during critical periods of development, we treated perinatal rat cortical neurons and astrocytes with increasing concentrations of CBD (0.1, 0.5, 1 or 5 µM) or a vehicle control, conducted a resazurin-based metabolic assay as well as LDH assay, and analyzed cell viability after 24 h of exposure (Figure 1A,B). We found evidence of a significant decrease in metabolic activity of neurons when exposed to 0.1 µM CBD and astrocytes when exposed to five-fold higher CBD concentration (Figure 1A). Moreover, CBD dose-dependently increased LDH efflux, which is a marker of cell death (Figure 1B). Treatment of neurons and astrocytes with the lowest effective CBD concentration (0.1 µM and 0.5 µM, respectively) for 1 to 24 h showed a rapid (after 2 h) decrease in astrocyte viability and a delayed yet significant decrease in neuronal viability (Figure 1C). Cell death as a function of time was confirmed by monitoring LDH release (Figure 1D). In contrast to our results, no reduction of cell viability was observed in the CTX-TNA2 rat astrocyte cell line after prolonged exposure (24 and 48 h) to CBD (range 1–1000 nM) [17] nor in the HT22 mouse hippocampal neuronal cell line after prolonged exposure (24 h) [18], nor perinatal rat cortical neurons after a brief exposure (i.e., 20–30 min) [19] to CBD at therapeutically relevant levels. However, CBD dose-dependently (range 0.1–10 µM, 20–30 min) induced cell death in rat optic nerve oligodendrocytes [19] and showed a toxic effect on perinatal rat hippocampal neurons [20] and murine microglial cells [21] at concentrations above 5 µM. Thus, the effects of CBD are not only concentration- and time-dependent but are likely to be cell-specific.

**Figure 2:**
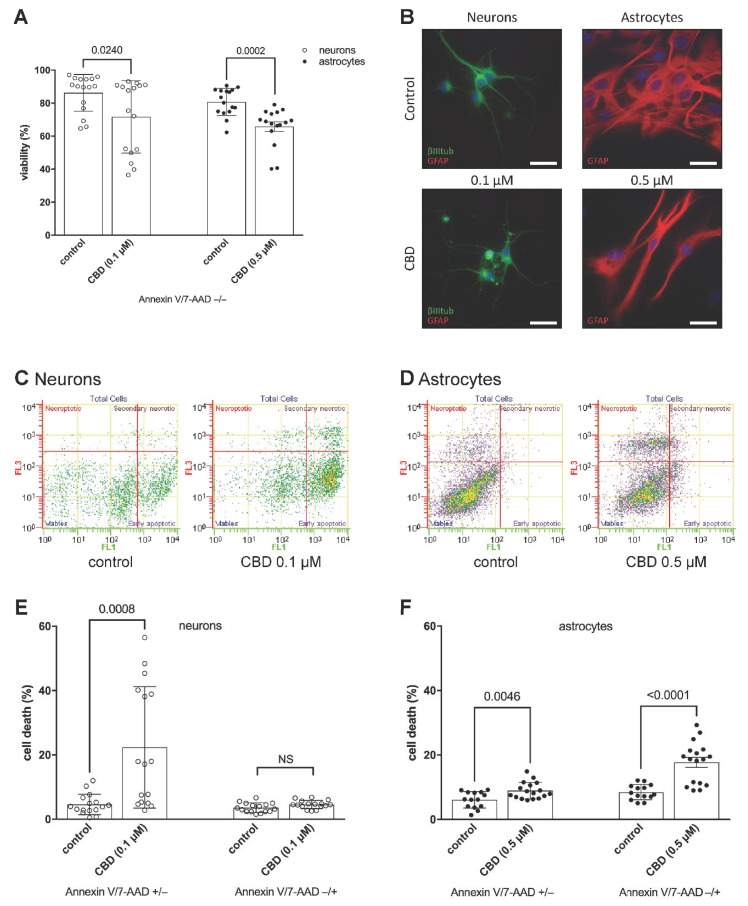
CBD reduces the viability of neurons and astrocytes by triggering various forms of cell death. (**A**,**C**–**F**) Neurons and astrocytes were incubated with 0.1 µM CBD and 0.5 µM CBD, respectively, for 24 h and then regenerated for an additional 22 h. (**A**) Viability of the cells was determined by flow cytometry, revealing percentages of overall cell viability (Annexin V/7-AAD −/−). Data were shown as mean ± SD. Independent sample *t*-test, *p* values vs. control were indicated. (**B**) Representative fluorescence images of the cultures after incubation with CBD or vehicle. Primary neurons were exposed to 0.1 µM CBD for 24 h and stained for neuron-specific β-III-tubulin (green), astrocyte-specific GFAP (red), and nuclear stain Hoechst (blue). Primary astrocytes were exposed to 0.5 µM CBD for 24 h and stained for GFAP (red) and nuclear stain Hoechst (blue). Scale bar = 30 µm. (**C**) Representative flow cytometry dot plot images, showing simultaneous binding of Annexin V (FL1) and 7-AAD (FL3) uptake by neurons after 0.1 µM CBD treatment. Control cells were not exposed to CBD. (**D**) Representative flow cytometry dot plot images, showing simultaneous binding of Annexin V (FL1) and 7-AAD (FL3) uptake by astrocytes after 0.5 µM CBD treatment. Control cells were not exposed to CBD. (**E**) Cell survival of neurons after 24 h exposure to 0.1 µM CBD and 22 h regeneration was measured by flow cytometry, revealing the percentages of early apoptotic cells (Annexin V/7-AAD +/−) and necroptotic cells (Annexin V/7-AAD −/+). Data were shown as mean ± SD. Independent sample *t*-test, *p* values vs. control were indicated. NS: not significant. (**F**) Cell survival of astrocytes after 24 h exposure to 0.5 µM CBD and 22 h regeneration was measured by flow cytometry, revealing the percentages of early apoptotic cells (Annexin V/7-AAD +/−) and necroptotic cells (Annexin V/7-AAD −/+). Data were shown as mean ± SD. Independent sample *t*-test, *p* values vs. control were indicated.

Indeed, despite approximately the same cytotoxic effect on neurons and astrocytes, leading to 14.6 ± 0.8% and 15.9 ± 0.8% cell loss, respectively (Figure 2A), flow cytometric analysis and morphology observation indicated differences in the mechanism of cell response to CBD. As cells can die in various ways [22], the double staining with Annexin V-FITC and 7-AAD makes it possible to distinguish cells in the early stages of apoptosis (Annexin V/7-AAD +/−) from those that die in a different manner (Annexin V/7-AAD −/+) in the same sample. A 24 h exposure to 0.1 µM CBD showed that perinatal cortical neurons underwent early apoptotic cell death, as Annexin V/7-AAD −/+ staining detecting necroptosis, a form of regulated necrosis [23], did not exceed control cells (Figure 2C,E). Acute exposure to CBD induces cytoskeletal disruption that contributes to the development of neurotoxicity [24]. By labelling neuronal β III tubulin and nuclei, we observed a reduction and shortening of dendrites, cell shrinkage, and chromatin condensation in neurons, all of which are characteristic morphological changes associated with apoptosis (Figure 2B). Astrocytes, however, responded differently. Following the 24 h exposure to 0.5 µM CBD, 8.9 ± 0.6% of cells (compared with 6.1 ± 0.7% control) were in the early apoptotic phase (Annexin V/7-AAD +/−), while 17.7 ± 1.6% of cells (compared with 8.5 ± 0.6% control) underwent necroptotic cell death (Annexin V/7-AAD −/+) (Figure 2D,F). The coexistence of various modes of cell death often occurs in a wide range of human pathologies [22,24]. Our data suggest that CBD could cause different morphological and biochemical changes in individual astrocytes, thus triggering different cell death pathways [22,23,24,25,26]. Astrocytes were shrunk with cytoplasmic vacuolation. Their protrusions were slanted, but their length did not appear affected. Examining the alignment of the specific intermediate filament GFAP revealed that the GFAP network is retracted toward the cell body and that the filaments in the protrusions are piled together (Figure 2B). The neurotoxicity of CBD on perinatal cortical neural cells may therefore be manifested at submicromolar concentrations, which cause the reorganization of the microfilament cytoskeleton along with suppression of cell viability and/or disruption of cell membrane integrity.

**Figure 3:**
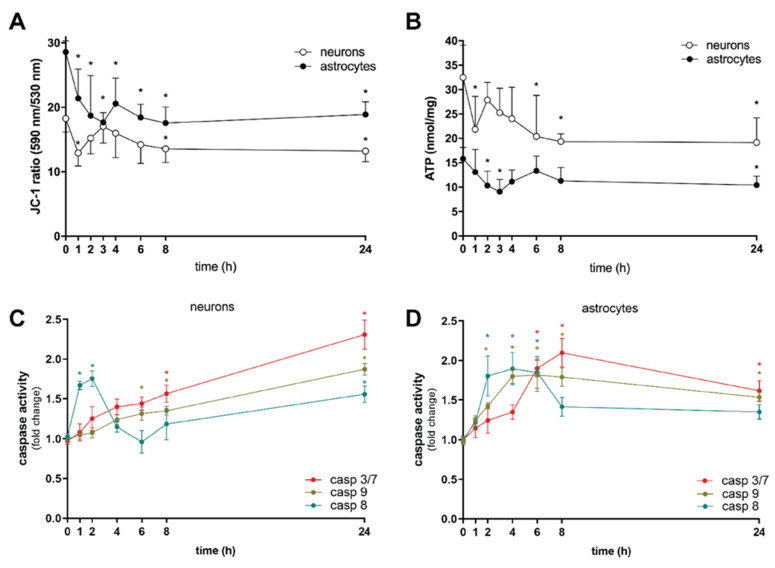
CBD impairs mitochondrial function, affects cellular energetic supply, and activates caspases in rat cortical neurons and astrocytes. Neurons were treated with 0.1 µM CBD, and astrocytes were treated with 0.5 µM CBD for 1 to 24 h. (**A**) Mitochondrial membrane potential was assessed with JC-1 dye; data (JC-1 fluorescence ratio) were shown as mean ± SD (*n* = 5). Two-way ANOVA (F_7,48_ = 6.516; *p* < 0.0001) followed by Tukey’s post hoc test, * *p* < 0.05 vs. control. (**B**) ATP levels were determined using ATPlite 1-step assay system; data were normalized to protein concentration and shown as mean ± SD (*n* = 5). Two-way ANOVA (F_7,63_ = 3.958; *p* = 0.0012) followed by Tukey’s post hoc test, * *p* < 0.05 vs. control. (**C**) Neurons were treated with 0.1 µM CBD for 1 to 24 h. Caspase activity was evaluated by Caspase-Glo 8, Caspase-Glo 9, and Caspase-Glo 3/7 assay; data were normalized to the mean value of the control and shown as mean ± SD (*n* = 5). One-way ANOVA, Dunnett’s post hoc test, * *p* < 0.05 vs. control. (**D**) Astrocytes were treated with 0.5 µM CBD for 1 to 24 h. Caspase activity was evaluated by Caspase-Glo 8, Caspase-Glo 9, and Caspase-Glo 3/7 assay, data were normalized to the mean value of the control and shown as mean ± SD (*n* = 5). One-way ANOVA, Dunnett’s post hoc test, * *p* < 0.05 vs. control.

As CBD promotes cell death, we subsequently investigated biochemical pathways that lead to brain cell dysfunction. CBD transiently disrupted membrane potential across the mitochondrial inner membrane (ΔΨm) (Figure 3A) and depleted ATP (Figure 3B) in neurons already during the first hour of exposure. The loss of ΔΨm that affects cellular energetic supply [27] was parallel to a transient increase of caspase-8 activation (Figure 3C), so the curious short-term change in neuronal ATP homeostasis may be also linked to the increased ATP consumption required to trigger the proapoptotic pathway [22]. This was succeeded by a phase of rapid partial recovery, during which sufficient ATP was presumably still present to progressively activate the initiator caspase-9. This cleaved and activated executioner caspases, resulting in eventual apoptotic death after 24 h of CBD exposure (Figure 3C). A different response to CBD was observed in astrocytes. Under our experimental conditions, dysregulation of ΔΨm was observed in the first hour of CBD treatment (Figure 3A), leading to transient ATP depletion over the next hour (Figure 3B) and concomitant activation of extrinsic and intrinsic caspases converging after 6 h to the level of executioner caspases, which reached the activity plateau two hours later (Figure 3D). Caspase activity then gradually decreased over time but remained significant even after prolonged exposure (24 h) to CBD. The parallel mechanism of transient activation of caspase-8 might be responsible for suppressing necroptosis [28,29], acting as a molecular switch that controls different modes of cell death (Figure 2), as previously documented in non-neuronal cells [30,31,32]. Therefore, the fast-triggered apoptosis may be succeeded by caspase-8 inhibition, which, over time, enables the interplay between apoptosis and necroptosis signalling in astrocytes. Thus, the neurotoxicity of CBD in perinatal cortical neurons and astrocytes probably involves not only intrinsic (caused by mitochondrial stress) but also extrinsic (death receptor-induced) proapoptotic pathways, with the latter being possibly of more significance in astrocytes.

**Figure 4:**
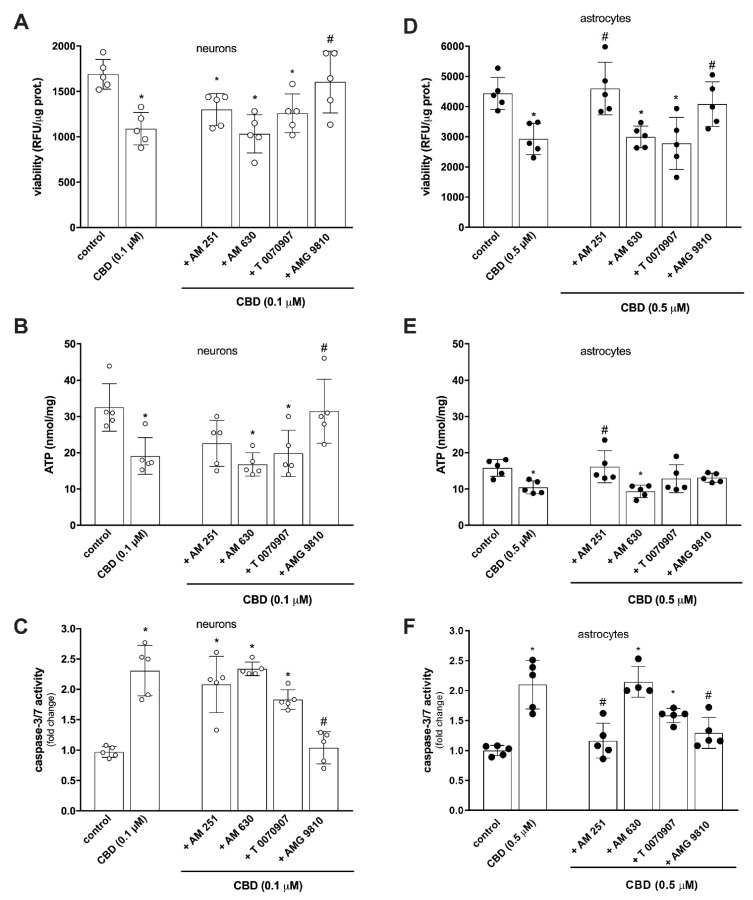
CBD decreases viability, affects cellular energetic supply, and activates caspases 3/7 in rat cortical neurons and astrocytes by TRPV1 and CB1/TRPV1 receptor activation, respectively. (**A**–**C**) Neurons were preincubated for 1 h with specific antagonists of CB1 (AM 251), CB2 (AM 630), PPARγ (T 0070907), or TRPV1 (AMG 9810) receptors, all at 0.1 µM, prior to 24 h challenge with 0.1 µM CBD. (**A**) Cell viability was evaluated by resazurin-based metabolic assay; data (relative fluorescence units—RFU) were normalized to protein concentration and shown as mean ± SD (*n* = 5). One-way ANOVA, Dunnett’s post hoc test, * *p* < 0.05 vs. control, ^#^ *p* < 0.05 vs. 0.1 µM CBD. (**B**) ATP levels were determined using ATPlite 1-step assay system; data were normalized to protein concentration and shown as mean ± SD (*n* = 5). One-way ANOVA, Dunnett’s post hoc test, * *p* < 0.05 vs. control, ^#^ *p* < 0.05 vs. 0.1 µM CBD. (**C**) Caspase 3/7 activity was evaluated by Caspase-Glo 3/7 Assay; data were normalized to the mean value of the control and shown as mean ± SD (*n* = 5). One-way ANOVA, Dunnett’s post hoc test, * *p* < 0.05 vs. control, ^#^ *p* < 0.05 vs. 0.1 µM CBD. (**D**–**F**) Astrocytes were preincubated for 1 h with specific antagonists of CB1 (AM 251), CB2 (AM 630), PPARγ (T 0070907), or TRPV1 (AMG 9810) receptors, all at 0.5 µM, prior to 24 h challenge with 0.5 µM CBD for viability and ATP evaluation or to 8 h challenge for caspase 3/7 evaluation. (**D**) Cell viability was evaluated by resazurin-based metabolic assay; data (relative fluorescence units-RFU) were normalized to protein concentration and shown as mean ± SD (*n* = 5). One-way ANOVA, Dunnett’s post hoc test, * *p* < 0.05 vs. control, ^#^ *p* < 0.05 vs. 0.5 µM. (**E**) ATP levels were determined using ATPlite 1-step assay system; data were normalized to protein concentration and shown as mean ± SD (*n* = 5). One-way ANOVA, Dunnett’s post hoc test, * *p* < 0.05 vs. control, ^#^ *p* < 0.05 vs. 0.5 µM CBD. (**F**) Caspase 3/7 activity was evaluated by Caspase-Glo 3/7 Assay; data were normalized to the mean value of the control and shown as mean ± SD (*n* = 5). One-way ANOVA, Dunnett’s post hoc test, * *p* < 0.05 vs. control, ^#^ *p* < 0.05 vs. 0.5 µM CBD.

Due to very low affinity and low agonist activity at canonical cannabinoid receptors [33], several non-canonical receptors, including peroxisome proliferator-activated receptor-γ (PPAR-γ) and transient receptor potential vanilloid 1 (TRPV1)—both targets of endogenous AEA [34,35,36]—have been identified to mediate CBD activity [12,37]. These receptors are dynamically and spatially positioned in the brain from early embryonic stages [37,38,39,40,41], with a prominent role in various developmental processes [7,40,41,42]. Pharmacological analysis revealed that under given conditions, only pre-treatment with TRPV1 antagonist AMG 9810 provided significant protection against compromised cell viability (Figure 4A), decrease in intracellular ATP concentration (Figure 4B), and activation of caspase 3/7 (Figure 4C). Therefore, it seems that TRPV1 is a possible molecular target mediating the action of CBD on perinatal neurons. TRPV1 is a stress response protein and a nonselective calcium-permeable polymodal cation channel [43]. It has a role in pain transmission, neurogenic inflammation, synaptic plasticity, neuronal overexcitability, and neurotoxicity [44,45,46]. Evidence from studies on cultured retinal [47] and trigeminal [48] ganglion cells, mesencephalic dopaminergic neurons [49,50] and cortical microglia [51], and rat hippocampal and cortical neurons [52,53] suggests that overactivation of TRPV1 by cannabinoid ligands may induce cell death via oxidative stress [52,54], mitochondrial disruption [55], and intracellular Ca^2+^ overload [43,52,56,57]. CBD is a full but less potent agonist of TRPV1 [37], binding the same specific site as capsaicin, a highly potent TRPV1-specific agonist [58]. Bisogno et al. [37] suggested that CBD is less capable than capsaicin at inducing a TRPV1-mediated functional response at low concentrations. However, our observation is in line with findings of other authors that confirmed the ability of capsaicin [50,52,53], endogenous AEA, and N-arachidonoyldopamine (NADA) to bind at micromolar concentrations functional TRPV1 expressed on cultured neurons [52] and cause Ca^2+^ influx, ERK phosphorylation, and reactive oxygen and nitrogen species production. Prolonged capsaicin treatment and thus prolonged activation of TRPV1 (24 h) increased the level of active caspase-3, followed by cell death. 

The detrimental effect of CBD on cell viability (Figure 4D) and induction of apoptosis (Figure 4F) in cortical astrocytes was effectively inhibited by targeting both TRPV1 and CB_1_ receptors. However, only the CB_1_ receptor antagonist AM 251, but not the TRPV1 antagonist AMG 9810, effectively reversed CBD-induced ATP depletion and thus maintained the energetic balance in the cells (Figure 4E). CBD has a low binding activity to CB_1_ receptor and due to its complex pharmacology can modulate the activity of several non-canonical receptors, transporters, and enzymes in addition to cannabinoid receptors [12]. The ECS is a fundamental effector of astrocyte function, with the crucial importance of CB_1_ receptor controlling complex processes such as synaptic, metabolic, and behavioral responses [59]. CB_1_ receptor is highly expressed in neurons. Its presence in astrocytes is barely detectable [60] and has been proven at the plasma membrane and at several subcellular locations, including mitochondria [61,62], adding complexity to the CB_1_-dependent regulation of astrocyte activity [59]. Recently reported findings demonstrated that prolonged exposure to Δ^9^-THC reduces glycolytic capacity and lactate release by engaging the astroglial mtCB_1_ receptor, resulting in neuronal bioenergetic stress and impaired social behavior [62]. Therefore, CBD-mediated signaling in cortical astrocytes affects the CB_1_ receptor, and perhaps the mtCB_1_ receptor, in addition to TRPV1, where their simultaneous expression allows for presently unknown functional interactions, leading to cell dysfunction.

This study could have clinical implications as we have demonstrated that CBD can injure perinatal brain cells in concentrations measured in patients with epilepsy taking CBD therapy [15]. Moreover, the lower level of CBD toxicity for perinatal cortical neurons was not established, since neurotoxicity was observed at the lowest tested concentration. It is interesting that clinical symptoms of CBD neurotoxicity have not yet been documented, but discrete symptoms could be easily overlooked in severely ill patients treated with high doses of CBD. At the moment, the only pharmaceutical preparation containing solely CBD is Epidiolex^®^ (Epidyolex^®^ in Europe), which is extracted directly from plants specifically grown to yield a high amount of CBD [63]. It is used for seizure control activity in patients with Dravet syndrome, without a fully elucidated mechanism of action [63]. According to PubMed search, this pharmaceutical preparation has not been used in published in vitro studies, only pure CBD provided by the producer. In future, the cytotoxic effects of CBD containing compositions should be evaluated as well. In summary, this study suggests caution in the use of CBD in humans, especially in pregnant women and children.

## 3. Conclusions

CBD impairs the function of perinatal rat cortical neurons and astrocytes at concentrations that are attainable in humans. Given that it is a non-intoxicating drug, our results clearly show that this is not the case during critical periods of brain development when CBD, as Δ^9^-THC [7,8], can significantly interfere with the endogenous cannabinoid system. By causing bioenergetic stress, reduced viability, and cell death, CBD may disrupt a delicate homeostatic balance between neural cells and contribute to developmental malfunction.

## 4. Materials and Methods

### 4.1. Materials

We purchased cannabidiol (CBD) (nat.) from Bionorica^®^ ethics (Neumarkt, Germany), and CB_1_ selective antagonist/inverse agonist AM 251, CB_2_ antagonist AM 630, PPARγ antagonist T 0070907, and TRPV_1_ antagonist AMG 9810 from Tocris Bioscience (Bio-Techne Corporation, Minneapolis, USA). CBD and antagonists were dissolved in ethanol absolute and diluted in culture medium prior to addition to the cells. The reagents and materials supplied with each kit are mentioned with each method.

### 4.2. Primary Cortical Neuron Culture 

Cultures of primary neurons were prepared from cerebral cortices of Wistar rat fetuses (E18-19) by the protocol approved by the Veterinary Administration of the Republic of Slovenia (No. U34401-3/2013/3). Cells were plated on poly-L-lysine (Sigma, Merck KGaA, Darmstadt, Germany) coated vessels and cultured in Neurobasal medium with 2% B27 Supplement, 0.5 mM GlutaMax, and 5 µg/mL gentamicin (all Gibco, Thermo Fisher Scientific Inc., Waltham, MA, USA). Glial proliferation was stopped by adding of 2 µM cytosine arabinoside (Sigma, Merck KGaA, Darmstadt, Germany) on day 3. Half of the medium was replaced every 3–4 days with fresh medium. Experiments were performed at 10–12 days in vitro.

### 4.3. Primary Cortical Astrocyte Culture

Cultures of primary astrocytes were prepared from cerebral cortices of 2-day-old neonatal Wistar rats by the protocol approved by Veterinary Administration of the Republic of Slovenia (No. 34401-87/2008/3) as previously described by Mele and Juric [64]. Cells were grown in DMEM/F12 (1:1), 10% (*v/v*) FBS, 100 U/mL penicillin, and 100 mg/mL streptomycin (all from Gibco BRL, Scotland, UK) and maintained in a humified atmosphere of 5% CO_2_ at 37 °C. After three overnight shakings, the monolayer cells were trypsinized, sub-cultivated, and then re-plated at an appropriate density in 6-well and 96-well culture plates (Nunc, Roskilde, Denmark) or poly-L-lysine-coated coverslips.

### 4.4. Treatment

Cultured cells were treated with a serum-free medium containing 0.1, 0.5, 1, or 5 µM CBD and incubated in a 5.0% CO_2_ humidified incubator at 37 °C for 1 to 24 h. Control cells were treated with an equivalent amount of vehicle (0.001% (*v*/*v*) ethanol). For inhibitor studies on neurons, cultured cells were preincubated for 1 h with 0.1 µM AM 251, AM 630, T 0070907, or AMG 9810 prior to 24 h challenge with 0.1 µM CBD. For inhibitor studies on astrocytes, cultured cells were preincubated for 1 h with 0.5 µM AM 251, AM 630, T 0070907, or AMG 9810 prior to 24 h challenge with 0.5 µM CBD. Antagonists did not affect control cells (data not shown).

### 4.5. Cell Viability Assays

#### 4.5.1. Metabolic Activity

Viability of cultured cells was quantified by monitoring metabolic activity with AlamarBlue^®^ redox indicator resazurin (Molecular Probes, Eugene, OR, USA) according to the manufacturer’s instructions. Fluorescence was quantified at the excitation and emission wavelengths of 540 and 595 nm, respectively, using a Synergy HT microplate reader (BioTek, Shoreline, WA, USA). Data (relative fluorescence units—RFU) were normalized to protein concentration determined by Bradford’s method [65] using BIO-RAD Protein Assay.

#### 4.5.2. LDH Release

Cell viability was determined by measuring LDH released using a CytoTox-ONE Homogenous Membrane Integrity Assay (Promega Corporation, Madison, WI, USA). The reaction mixture was added to the cells, and fluorescence was quantified at the excitation and emission wavelengths of 560 and 590 nm, respectively, using a Synergy HT microplate reader (BioTek, Shoreline, WA, USA).

#### 4.5.3. Flow Cytometry Detection of Cell Death

Cell death was determined after double staining of the cells with Annexin V-FITC and 7-Aminoactinomycin D (7-AAD) staining kit (Beckman-Coulter, Brea, CA, USA), which allows the determination of viable cells (Annexin V/7-AAD −/−), the fraction of early apoptotic cells (Annexin V/7-AAD +/−), the cells in necroptosis (Annexin V/7-AAD −/+), and late apoptotic and necrotic cells (Annexin V/7-AAD +/+). The cells were stained according to the manufacturer’s instructions. Briefly, cells were centrifuged at 500 rcf at 4 °C for 5 min and washed with cold PBS. Then, the cells were resuspended in 100 µL of cold 1x binding buffer and stained with 10 µL of Annexin V-FITC and 20 µL of 7-AAD solution. After staining, the mixture was incubated in the dark at 4 °C for 15 min. After incubation, an additional 400 µL of 1× binding buffer was added. Data acquisition was carried out in a Quanta SC MPL flow cytometer (Beckman Coulter, Brea, CA, USA). Every measurement includes 10,000 cells.

### 4.6. Change in Mitochondrial Membrane Potential (ΔΨm)

Cells were subjected to several treatments, incubated in the presence of the JC-1 probe (Cayman Chemical Company, Ann Arbour, USA) (5 μg/mL) at 37 °C for 15 min, and then washed twice with Assay Buffer. Fluorescence was detected on a Synergy HT microplate reader (BioTek, Shoreline, WA, USA). The ratio of fluorescence intensity of JC-1 aggregates to monomers was used as an indicator of mitochondrial membrane potential.

### 4.7. ATP Measurement

Intracellular ATP concentration was determined using an ATPlite 1-step assay system (Perking Elmer, Boston, MA, USA), which generates a luminescence signal proportional to the amount of ATP present [66]. ATP content was calculated using a generated standard ATP curve (1 pM–1 µM) and normalized to the protein concentration determined by Bradford’s method [65].

### 4.8. Detection of Caspase Activity

Following CBD exposure, the cells were harvested and solubilized in a cell culture lysis buffer. The activity of caspase-8, caspase-9, and caspases-3/7 was determined from the formation of luminescent substrates using Caspase-Glo 8, Caspase-Glo 9, and Caspase-Glo 3/7 Assay (Promega, Madison, WI, USA).

### 4.9. Immunocytochemistry

Cells grown on coverslips were rinsed with PBS, fixed in 4% paraformaldehyde (Sigma, Merck KGaA, Darmstadt, Germany) for 15 min, and permeabilized with 0.1% Triton-X for 5 min at room temperature. The coverslips were rinsed and then incubated overnight at 4 °C with rabbit anti-β III tubulin (1:500, Abcam, Cambridge, UK) and/or mouse anti-glial fibrillary acidic protein (GFAP) (1:500, Millipore, Burlington, MA, USA). The next day, coverslips were rinsed and incubated with corresponding Alexa Fluor 488 and 546 conjugated secondary antibodies (1:500, Molecular Probes, Eugene, OR, USA) for 1 h at 37 °C. The nuclei were labelled by Hoechst (2 µg/mL, Molecular Probes, Eugene, OR, USA) for 10 min at 37 °C. Following the washing, the coverslips were mounted in ProLong Diamond (Molecular Probes, Eugene, OR, USA) and observed under the fluorescence microscope (Olympus, Tokyo, Japan).

### 4.10. Data Analysis

All data are presented as mean ± SD from five independent experiments (*n* = 5) carried out in triplicate. Data analysis was performed using GraphPad Prism version 9.3.0 (GraphPad Software, San Diego, CA, USA, www.graphpad.com) (accessed on 2 November 2021). The statistical significance of differences between the values was evaluated by analysis of variance (ANOVA) followed by Tukey’s or Dunnett’s post hoc test for multiple-group comparisons or independent sample *t*-test. A *p* value of <0.05 was considered to be statistically significant.

## Data Availability

The data that support the findings of this study are available from the corresponding author, M.B., upon reasonable request.
